# Mapping Seabird Sensitivity to Offshore Wind Farms

**DOI:** 10.1371/journal.pone.0106366

**Published:** 2014-09-11

**Authors:** Gareth Bradbury, Mark Trinder, Bob Furness, Alex N. Banks, Richard W. G. Caldow, Duncan Hume

**Affiliations:** 1 Wildfowl & Wetlands Trust (Consulting) Ltd., Slimbridge, United Kingdom; 2 MacArthur Green, Glasgow, United Kingdom; 3 Natural England, Exeter, United Kingdom; 4 Marine Management Organisation, Newcastle, United Kingdom; Centro de Investigacion Cientifica y Educacion Superior de Ensenada, Mexico

## Abstract

We present a Geographic Information System (GIS) tool, SeaMaST (Seabird Mapping and Sensitivity Tool), to provide evidence on the use of sea areas by seabirds and inshore waterbirds in English territorial waters, mapping their relative sensitivity to offshore wind farms. SeaMaST is a freely available evidence source for use by all connected to the offshore wind industry and will assist statutory agencies in assessing potential risks to seabird populations from planned developments. Data were compiled from offshore boat and aerial observer surveys spanning the period 1979–2012. The data were analysed using distance analysis and Density Surface Modelling to produce predicted bird densities across a grid covering English territorial waters at a resolution of 3 km×3 km. Coefficients of Variation were estimated for each grid cell density, as an indication of confidence in predictions. Offshore wind farm sensitivity scores were compiled for seabird species using English territorial waters. The comparative risks to each species of collision with turbines and displacement from operational turbines were reviewed and scored separately, and the scores were multiplied by the bird density estimates to produce relative sensitivity maps. The sensitivity maps reflected well the amassed distributions of the most sensitive species. SeaMaST is an important new tool for assessing potential impacts on seabird populations from offshore development at a time when multiple large areas of development are proposed which overlap with many seabird species’ ranges. It will inform marine spatial planning as well as identifying priority areas of sea usage by marine birds. Example SeaMaST outputs are presented.

## Introduction

As the intensity and scale of offshore renewable energy development reaches unprecedented levels with the third round of offshore seabed leasing in UK waters underway [1], there is a need for careful examination of potential impacts to seabird populations, some of which are already under pressure [2]. As well as informing impact assessment for current proposals, it is timely that understanding of ornithological spatial sensitivity to such developments is explored, as marine spatial plans for England are currently being drawn up [3]. These plans will be used to inform identification of areas suitable for future development with the aim of minimising consenting risk by avoiding areas of highest sensitivity.

Including such diverse and biologically productive areas as Dogger Bank, Farne Deep, the Outer Thames Estuary and much of Liverpool Bay, English territorial waters provide important foraging for breeding, wintering and passage seabirds, seaducks, divers and grebes. Twenty-two of the UK’s 25 species of breeding seabirds nest within range of these productive areas on England’s offshore islands, mainland cliffs and beaches. Many of these breeding sites are protected under the Birds Directive (2009/147/EC) as Special Protection Areas (SPAs). These sites provide for most of the UK’s breeding black-headed gulls *Chroicocephalus ridibundus*, Mediterranean gulls *Larus melanocephalus*, lesser black-backed gulls *Larus fuscus*, little terns *Sterna albifrons*, Sandwich terns *Sterna sandvicensis* and roseate terns *Sterna dougallii* [4]. There are additional internationally important aggregations of other seabirds including several auk species, other terns and gulls, storm petrels *Hydrobates pelagicus*, European shags *Phalacrocorax aristotelis,* great cormorants *Phalacrocorax carbo* and northern gannets *Morus bassanus*. In the non-breeding season (UK winter), Liverpool Bay SPA and the Outer Thames Estuary SPA support the UK’s largest populations of overwintering common scoters *Melanitta nigra* and red-throated divers *Gavia stellata* respectively [5] [6]. A further potential site in Cornwall (Falmouth Bay to St Austell Bay pSPA) for inshore divers and grebes represents the beginning of the next wave of proposed marine protection in England.

As wide-ranging, long-lived birds with delayed sexual maturity and low annual productivity, many species of seabirds and coastal waterbirds are potentially at risk to impacts from offshore wind farms [7]. The main risks are from fatal collision with turbines; displacement from the wind farm area due to disturbance; barrier effects; and habitat loss [8] [9], although sensitivity assessments have not tended to focus on the latter two factors as these tend to apply in a case-specific rather than generic fashion. As empirical data on the effects of specific anthropogenic impacts on seabirds are generally unavailable, previous studies, for example with regard to oil pollution and the sandeel fishery, have adopted approaches that produce relative sensitivity scores and rankings of seabirds to such impacts. Following the approach of King & Sanger [10], Williams *et al.* [11] and Furness & Tasker [12], Garthe and Hüppop [13] assigned Species-specific Sensitivity Indices (SSIs) to seabirds based on aspects of their life history, behaviour and status which made their populations more or less vulnerable to the risks of collision and displacement from wind farms. By combining the SSIs with densities of birds at sea, Garthe and Hüppop produced relative vulnerability maps for German North Sea waters. Furness *et al.* [14] subsequently revised the SSI approach for seabirds in Scottish waters, extending the species list considered and separating the assessments of risk due to collision from those of displacement or avoidance as this better identified those species which were particularly sensitive to one impact but not the other.

Recent developments for modelling seabird distributions such as Density Surface Modelling (DSM) [15], make use of both one dimensional (e.g. water depth, distance to shore) and two dimensional covariates (e.g. spatial location) in a Generalised Additive Modelling framework (GAM) [16] [17]. DSM also allows correction for imperfect detection of data collected using line transect methods (e.g. by visual observers from boats or aircraft [18]).

In summary, the aims of this study were to: produce SSIs for seabirds relevant to English waters; use DSM to map densities of seabirds in English territorial waters; and to combine the two outputs to produce seabird sensitivity layers in a GIS tool for use by all associated with both the offshore wind farm industry and marine spatial planning in England.

The GIS tool produced, SeaMaST (Seabird Mapping and Sensitivity Tool), is already being used for these purposes and for improving the evidence base upon which impact assessment advice and regulating work is founded. It provides a model for sensitivity analyses of other sectors potentially impacting seabirds, and sets out an approach that will be of relevance to others engaged in marine spatial planning and impact assessment in other countries requiring management of marine areas for seabirds.

## Methods

### Seabird datasets

The study area included all marine waters under English jurisdiction – from the mean low-water mark of the English coast out to a maximum of 200 nautical miles or the neighbouring territorial waters boundary. The two main datasets used were the European Seabirds at Sea (ESAS) database (maintained by the Joint Nature Conservation Committee (JNCC) [19]) and visual aerial survey data collected by Wildfowl & Wetlands Trust (Consulting) Ltd (WWT Consulting) under contract to statutory nature conservation agencies and offshore wind farm developers. The ESAS database included over 310,000 seabird records within the area of interest, from 1979 to 2011. The records were predominantly from boat-based surveys using 300 m wide line transects (see Camphuysen *et al.* [20]).

The WWT Consulting database contained over 400,000 seabird records from visual aerial surveys between 2001 and 2011 using a consistent line transect methodology [21].

Data collected during boat surveys for offshore wind farms where publically available through the Crown Estate Data Catalogue [22] were also included if appropriate effort and observation data were provided. Some offshore wind survey data were available from as recently as 2013.

### Seabird data processing

DSM requires the line transect survey data to be divided into discrete segments each with their own distance-corrected seabird abundance as well as values for candidate explanatory covariates. Any relationships thus identified between seabird abundance and covariate values can then be used to predict seabird abundance in areas not surveyed (e.g. between transects and outside survey areas).

Segments for ESAS data were taken as the recording periods, usually 10 minute time intervals, for which a midpoint location was either provided in the ESAS dataset or calculated.

Unlike the ESAS boat surveys, the WWT Consulting aerial surveys and more recent wind farm boat surveys employ continuous recording. These data were thus segmented for analyses, taking approximately 1 km segment lengths [16].

Boat and aerial segment covariate files were populated with the following covariates; minimum distance from coast, mean depth (from a 6 arc second Digital Elevation Model), x (easting in OSGB datum) and y (northing in OSGB datum) of the midpoint of each segment.

### Seabird data analysis

For this project we used parameter estimates obtained in DSM to predict densities of seabird species in 3 km×3 km mapping grid cells. DSM was developed to enable modelling of line transect data across regions with varying survey coverage and with overdispersed, zero-inflated data [17]. The analyses also produce measures of confidence in abundance and density estimates, which are useful in outputs to be used in decision making. DSM is now an established technique for the analysis of seabird survey data, allowing rigorous model testing and refinement (Burt *et al.* [23], Petersen & Nielsen [24], Rexstad & Buckland [25] and Bradbury *et al.* [26]).

To account for the complex coastline included in the study area, soap film smoothing was added to the DSM [27] [28] [15]. This fits a series of ‘knots’ over the data and treats the boundary as a fixed obstacle that predictions cannot transgress. In trials this proved effective at preventing nonsensical predictions of higher density areas across headlands, such as the Cornwall peninsula.

DSM as originally conceived is based on GAMs which are unable to account for the presence of spatial autocorrelation. However, since autocorrelation is a common feature of spatial ecology data it was necessary to consider alternative models on which to base inference. Mixed models allow the inclusion of random effects which can account for spatial autocorrelation. Unfortunately at the time of analyses, soap film smoothing methods could not be combined with Generalised Additive Mixed Models (GAMMs). As the inclusion of a soap film smooth had proved useful in initial (GAM based) trials, it was considered that this aspect was more important to retain than being able to account for spatial autocorrelation. However, to investigate the possible effects of omitting autocorrelation, a comparison of outputs from GAMMs including segment IDs as random effects and GAMs without the random effect were produced for species in Liverpool Bay, using only the spatial covariates x and y. The general seabird distributions were very similar between the two models, but the GAM model produced a slightly more flexible fit and lower Coefficients of Variation (CVs), as would be expected when modelling correlated data using a model which assumes no autocorrelation was present. Thus, by favouring a realistic spatial constraint (soap-smoothing) over correction for auto-correlation, the outputs discussed here are expected to have slightly overestimated precision; the CVs should be viewed as minima.

As the first stage of DSM, the boat and aerial line transect datasets were corrected for detectability of species. Distance analysis was performed using package Distance [29] in the statistical software ‘R’ [30] to produce detection functions for each species using the methods of Buckland *et al.* [31]. Typically this is carried out for each survey; however, given the large number of surveys to be analysed we pooled detection functions for each species in each dataset, analysing sitting and flying records separately with all records split further into summer and winter seasons (summarised as April – September and October – March respectively). ESAS surveys which did not record all species were only used as appropriate (i.e. auk only surveys were excluded for all but analysis of auk data) to prevent over-estimating effort. Flying birds recorded on strip transects were assumed to have a detection probability of 1 [20]. Pooling data had the benefit of increasing robustness where small sample sizes may have skewed estimated detection functions. Furthermore, this boosted sample sizes since flying birds often had no distance recorded (and indeed methods for assigning distance to such individuals are not standardised). Candidate detection functions were hazard-rate and half-normal with selection by Akaike’s Information Criteria (AIC) scores. Binned sea state (0, 1–3, 4–6) was included as a covariate for fitting detection functions in trials, but was found to add little to models (including higher AIC scores) so was removed from subsequent analyses. Species with fewer than 50 observations (either sitting or flying) were excluded from detection function fitting, but were made available in the SeaMaST datasets so locations of individual records of these rarer species could be mapped.

For those species with sufficient records for distance analysis but where density surface models could not be fitted because data were too sparse, the detection function was applied and relative densities produced by normalising by coverage in each 3 km×3 km grid cell, i.e. using no further modelling.

Minimum distance to coast (cdist) of the midpoint of each segment was populated in each database covariate file using geoprocessing in Esri ArcGIS 10 and mean depth was assigned using geoprocessing of a six arc second digital elevation model supplied by the UK Hydrographic Office under licence to Natural England. The fitting of GAMs/GAMMs was carried out in the ‘R’ package dsm [29] and mgcv [32].

Prior to DSM the databases were split into two regions – Liverpool Bay, which in this study was discreet, as Welsh territorial waters south of Liverpool Bay were not included, and the remaining English territorial waters stretching from the Bristol Channel around to Northumberland.

The function vif in the ‘R’ package HH [33] was used to check colinearity between the covariates. The Variance Inflation Factors (VIF) showed that depth and cdist had high covariances for most species/behaviour/seasons in Liverpool Bay but when covariates were compared for explanatory power cdist had higher power and was thus used preferentially.

‘x’, ‘y’ and cdist were added as covariates and for soap film models a series of knots fitted over the data using the function cover.design in the package ‘fields’ [34]. The number of soap knots was a compromise between effective data sampling and processing time required, with 30 eventually used and ‘k’ set to 10 based on visual assessment of outputs. Due to the large number of data combinations to be analysed, the process was automated in ‘R’ scripted loops. If the cover.design function located knots on the study area boundary the soap film smooth would fail, so the cover.design and soap film modelling was looped to try 10 attempts, and if that failed a model without soap film was used. This occurred only in WWT aerial data in the non-Liverpool Bay area for some analyses of particularly coastal species.

GAM model selection was by Restricted Maximum Likelihood (REML) with automatic term selection based on adding penalties to successive terms [35]. The error distribution family used was negative binomial; however on reviewing the results for common scoters, the model statistics and predicted densities showed a poor fit in the larger English waters area. Consequently the Tweedie distribution family was used for this species which resulted in more realistic predictions.

Density predictions were made on the 3 km×3 km prediction grid with area 9 km^2^ (or less for coastal cells) as the offset for abundance estimates. These were subsequently converted to densities expressed as birds/km^2^ for mapping.

Variance estimation used the dsm.var.gam function of package dsm [29], where variances were calculated using Bayesian interpretation of the GAM [36]. Using this method, uncertainty in the detection function was included using the delta method [31]. The Variance Propagation method of Williams *et al.* [28] was trialled, however at the time of analyses the dsm.var.prop function in package dsm could not handle GAMs with soap film smooths. Trials on data without soap film smooths showed the two variance estimation methods to produce almost identical results.

The estimated variances produced by dsm.var.gam were converted to CVs in each prediction grid cell for mapping.

### Species sensitivity to wind farms

The method used for scoring marine bird sensitivity to collision with wind turbines and avoidance or displacement from wind farm areas followed Furness and others [37] [14], amended for relevance to English waters and using updated data where this had become available. This method identified factors representing conservation importance and aspects of species vulnerability to these wind farm impacts. As far as possible, the scoring criteria for each factor and the respective provisional scores for each marine bird species were evidence-based, with data taken from reviewed literature. Four factors representing conservation importance and six representing aspects of species’ behaviour that influence their vulnerability to wind farms were used. The conservation importance factors were: status in relation to the Birds Directive; percentage of the biogeographic population that occurs in England/English waters during any particular season (taking account of turnover of individual birds); adult survival rate; and UK threat status. Status in relation to the Birds Directive scored Annex 1 species as 5, migratory birds which are features of SPAs as 3, and other marine species as 1, as in Furness and Wade [37]. Note that splitting Annex I and other migratory species identifies conservation priorities, but does not differentiate these species in terms of status, as each is offered equal protection. Here it is used as a proxy measure of conservation ‘importance’.

Percentage of the biogeographic population that occurs in England/English waters during any particular season scored 5 for species with more than 20% of the biogeographic population occurring in English waters at some period during the year, 4 for species with 10–19.9%, 3 for species with 5–9.9%, 2 for species with 1–4.9%, 1 for species with less than 1% of the biogeographic population in English waters at any time of year. For adult survival rate scores, published data on adult survival rate were used as a measure of the position of each species on the ‘r-K continuum’ which reflects the vulnerability of species to any increase in mortality above natural mortality (species with low adult survival rates tending to have early age of first breeding and high reproductive output and so be less vulnerable to additional mortality than the extreme ‘k-selected’ species). Data were taken from Garthe and Hüppop [13], Saether [38], del Hoyo *et al.* [39] [40], Glutz von Blotzheim and Bauer [41], or estimated from data for closely related species. Where several estimates were available, preference was given to more recent studies, and those from the UK, since species-specific survival rates may sometimes differ between geographical regions. Adult survival rates were classified on a 1 to 5 scale following the banding used by Garthe and Hüppop [13]: 1 (adult survival less than 0.749), 2 (adult survival 0.75–0.799), 3 (0.80–0.849), 4 (0.85–0.899) and 5 (adult survival above 0.90). UK Conservation Status factor reflected both threat and conservation status of the species in the UK, as given by Eaton *et al.* [42] in ‘Birds of Conservation Concern 3′ (BOCC3). For some species, the classification in BOCC3 differs from that in the previous assessment (BOCC2), and these changes are also taken into account here, given the implications of changes in status. Scores were allocated as follows: 1 (green in BOCC2 and BOCC3), 2 (amber in BOCC2 and green in BOCC3), 3 (green in BOCC2 and amber in BOCC3), 4 (amber in BOCC3 and BOCC2), and 5 (red in BOCC3). Other combinations could theoretically have occurred but were not represented in the data set. Weightings could have been given to these different scores, but it is unclear that particular scores should be more important than others.

The species vulnerability factors used were: flight altitude, flight manoeuvrability, percentage of time flying, nocturnal flight activity, disturbance by wind farm structures, ship and helicopter traffic, and habitat specialisation. Scores were assigned on a scale of 1 to 5 for almost all factors, where 5 was a strong anticipated negative impact. However, it was felt more appropriate to score flight altitude as percentage of a species’ flight altitude spent at turbine blade height (defined generically as approximately 20 to 150 m asl), rather than on a five point scale (as done by Furness and Wade [37]) but differing from the approach used by Garthe and Hüppop [13]). Flight manoeuvrability took into account the aerial agility of species and hence their potential to avoid collision with wind turbines at sea (a behaviour related to ‘microavoidance’ which assesses the ability of birds to avoid turbines at close range, but quite separate from ‘macroavoidance’ where birds may simply alter flight path to avoid coming close to turbines). Following Garthe and Hüppop [13], we assumed that, all other factors being equal, birds with low flight manoeuvrability were more likely to collide with wind turbines at offshore wind farms than birds with high flight manoeuvrability. Scores were taken from Garthe and Hüppop [13], but adjusted where more recent data were available. For additional species, scores were based on peer-reviewed literature. Species were classified from ‘very high flight manoeuvrability’ (score 1) to ‘very low manoeuvrability’ (score 5). Percentage of time flying indicated the risk of collision as marine birds that spend more time flying while at sea (whether while breeding, migrating, wintering, or as pre-breeders) are more likely to be at risk of collision. Where available, scores were taken from Garthe and Hüppop [13] and adjusted where more recent data suggested appropriate. For other species, scores were calculated from data on activity budgets following the procedure outlined by Garthe and Hüppop [13]. Species were scored 1 if 0–20% of time at sea was spent in flight, 2 if 21–40% was spent flying, 3 if 41–60% was spent flying, 4 if 61–80% was spent flying, and 5 if 81–100% was spent flying. This factor will probably vary seasonally, with the literature indicating more flight activity while rearing chicks than during the incubation period, and more flight while breeding than during winter. Peaks of flight activity occur in migrant species during the migration, while flight activity may be reduced during post-breeding moult. However, these refinements were not yet well enough documented to assess scores separately for different seasons, although that could be a useful future development of the method. For nocturnal flight activity we used scores published in Garthe and Hüppop [13] for the species where these were available: Score 1 (limited flight activity at night) to score 5 (much flight activity at night). For additional species we used published data where possible, and information (often qualitative rather than quantitative) from individual species studies or from handbooks [43] [39] [40] [41]. There was insufficient information on changes in flight behaviour (e.g. flight heights) of individual species to take such changes into account here; the assumption being made that such changes did not vary dramatically between species.

Again, following Furness and Wade [37] but differing from the approach used by Garthe and Hüppop [13], individual factor scores were combined to give a total for each species that ranked species according to their vulnerability to offshore wind farm developments separately in terms of collision risk and habitat loss through avoidance. Disturbance by wind farm structures, ship and helicopter traffic factor used scores from 1 (limited escape behaviour and a very short flight distance when approached), to 5 (strong escape behaviour, at a large response distance) again taken from Garthe and Hüppop [13] where available and adjusted where more recent data were available (e.g. Schwemmer *et al.* [44]). For additional species, information on disturbance sensitivity was taken from peer-reviewed literature. The habitat specialisation factor represents the range of habitats species are able to use and whether they use these as specialists or generalists. This score classifies species into categories from 1 (tend to forage over large marine areas with little known association with particular marine features) to 5 (tend to feed on very specific habitat features, such as shallow banks with bivalve communities, or kelp beds). Where available, scores presented by Garthe and Hüppop [13] were used. Scores for other species were based on foraging ecology described in single species studies in the literature, or from standard handbook descriptions.

Garthe and Hüppop [13] calculated species vulnerability scores using flight altitude (*a*), flight manoeuvrability (*m*), percentage of time flying (*t*), nocturnal flight activity (*n*), disturbance by wind farm structures, ship and helicopter traffic (*d*), and habitat specialisation (*h*) according to Equation 1:
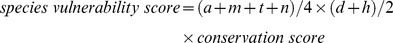
(1)


This recognised that the first four factors all related to flight ability and flight behaviour, while the last two factors relate to habitat use and susceptibility to disturbance. Thus their index combined both collision risk and disturbance/habitat loss considerations into a single score.

We used an alternative approach and scored separately for collision risk and for disturbance/habitat displacement risk. For collision risk, we gave a high weighting to flight altitude (*a*), and lower weighting to manoeuvrability (*m*), percentage of time flying (*t*), and nocturnal flight activity (n) (Equation 2).
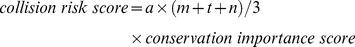
(2)For disturbance/habitat displacement we calculated a vulnerability index according to equation 3 where *d* and *h* represent disturbance by wind farm structures, ship and helicopter traffic, and habitat specialisation respectively. Note the two resulting scales should not be compared in a quantitative way but only in terms of the species ranking within one scale. 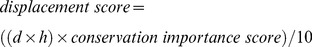
(3)


### Wind farm sensitivity mapping

For species sensitivity mapping the sensitivity to wind farm collision and displacement scores were applied to a function of the density of those species in each 3 km×3 km grid cell across the study area. As with previous studies [13] [45] the natural logarithm of the density has been used as it enabled better scaling for comparison between species and areas. So for each species’ sensitivity to wind farm impacts the expression took the form:
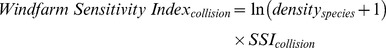
(4)

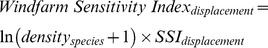
(5)Where SSI is the Species Sensitivity Index to either wind farm collision or displacement.

Overall sensitivity to wind farms in each 3 km×3 km grid cell were derived by assigning categorical scores to the ranked species sensitivity lists and summing the highest values for either of collision or displacement risk. Scores of 5 were assigned to those at ‘Very High Risk’, 4 to ‘High Risk’, 3 to ‘Moderate Risk’, 2 to ‘Low Risk’ and 1 to ‘Very Low Risk’. Note, however that the top rank ‘Very High Risk’ was not assigned for displacement concern, acknowledging the lower risk to populations compared to collision risks.

Thus for each species overall sensitivity to wind farm impacts the expression took the form:
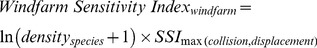
(6)


Densities for each species in each season (summer or winter) were produced by combining sitting and flying density estimates produced from either spatial modelling or corrected count estimates (for rarer species) from the boat and aerial datasets. The datasets were then compared and for each cell the maximum density value taken for that species and season if the CV for that analyses was less than 0.3, otherwise the density value with the lowest CV was selected. A threshold CV of 0.3 was chosen as reference to the predicted density maps, coverage maps and species observations showed areas with good coverage tended to have density predictions with associated CVs of 0.3 or lower. Similarly predicted densities with associated CVs over 0.5 were not included as these were found mainly in areas at greater distances from coverage and were deemed unreliable. For example this excluded predicted densities from inshore aerial surveys data in further offshore areas. Using this method, combined density maps were produced for each species for each season. To these the collision, displacement and overall sensitivity scores for each species were applied as per Equations 4, 5 and 6, and these summed to produce overall spatial sensitivity maps for collision, displacement and collision and displacement combined for each season, as per:
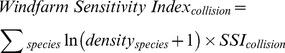
(7)

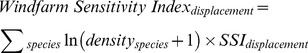
(8)

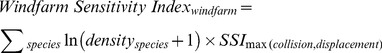
(9)


Esri ArcGIS 10 was used to perform final geoprocessing and to collate outputs as GIS layers to be included in SeaMaST.

## Results

**Figure 1:**
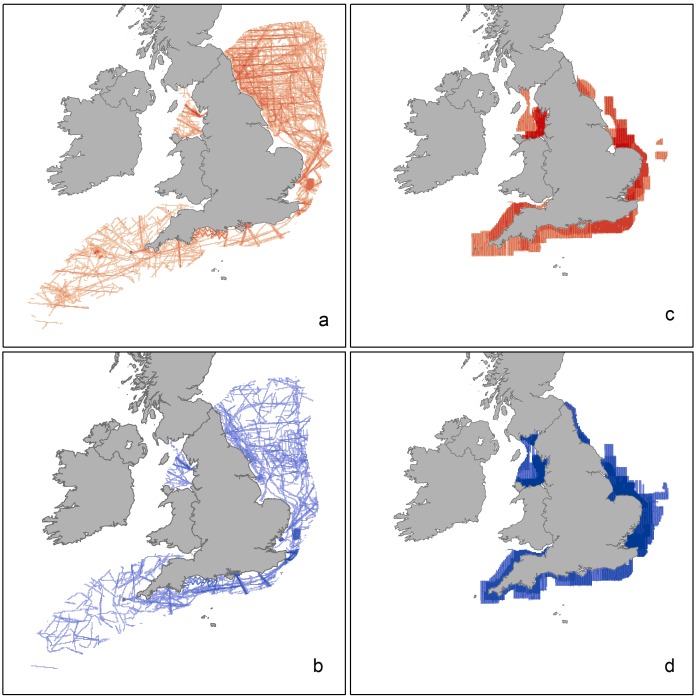
Survey effort as area (km^2^) in each 3 km×3 km cell of the prediction grid covering the Seabird Sensitivity Mapping in English Waters study area. a) JNCC European Seabirds at Sea (ESAS) boat surveys in summer months (April to September inclusive) from 1979 to 2011 and b) in winter months (October to March inclusive). c) WWT Consulting aerial surveys in summer months (April to September inclusive) from 2001 to 2011 (excluding Round 3 wind farm data) and d) in winter months (October to March inclusive).

**Figure 2:**
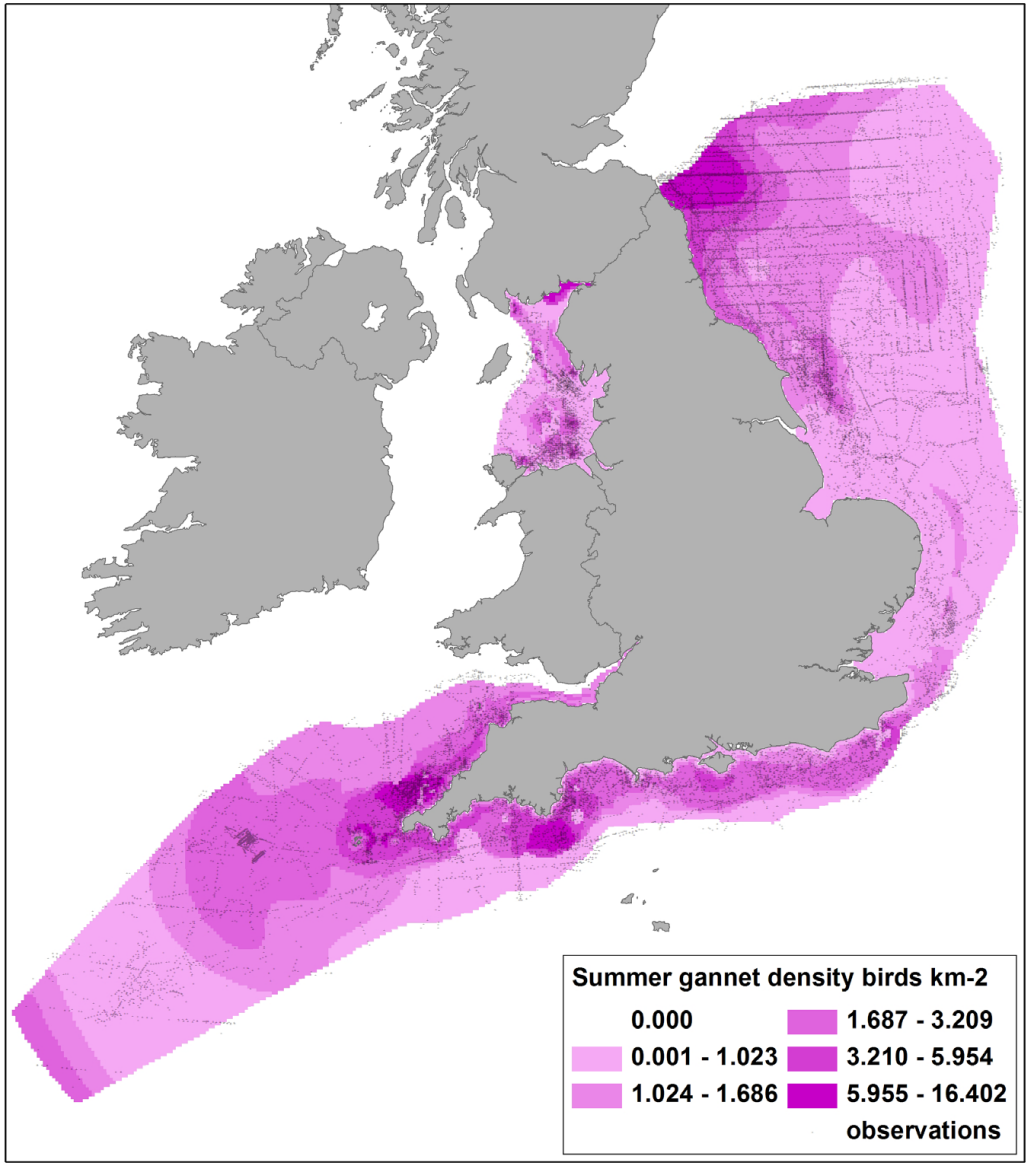
Predicted densities of gannets in summer from DSM of ESAS boat and WWT Consulting aerial survey data, with survey observations shown. DSM used *x*, *y* and *cdist* covariates and a soap film smooth. Note the observations do not take account of coverage so seemingly high use areas may still have low predicted densities if coverage was also high.

**Figure 3:**
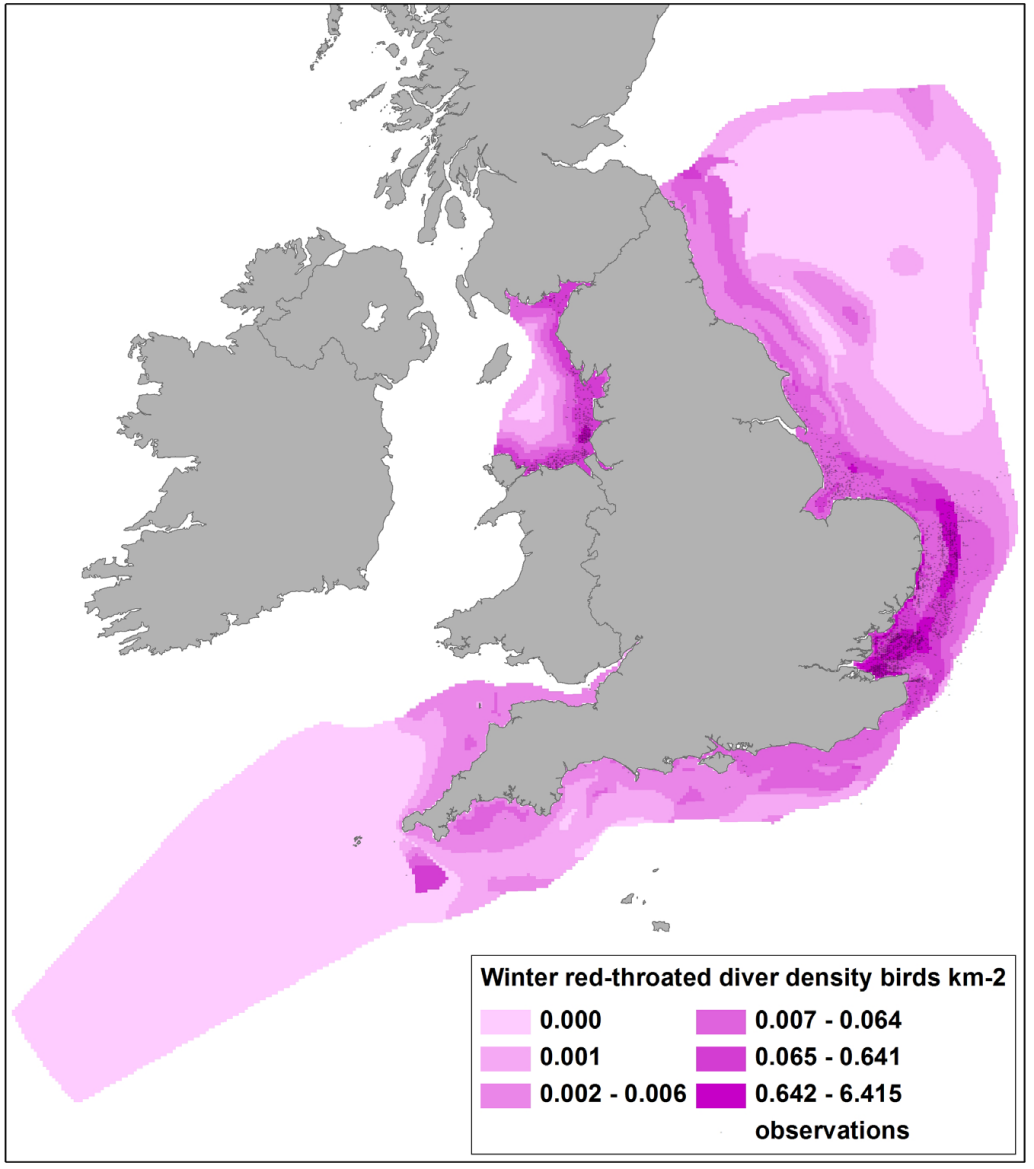
Predicted densities of red-throated divers in winter from DSM of ESAS boat and WWT Consulting aerial survey data, with survey observations shown. DSM used *x*, *y* and *cdist* covariates with a soap film smooth used only in Liverpool Bay due to the difficulty in locating knots very close to the coast. Note that the model produced unfeasible predictions further offshore where there was no aerial survey coverage which provided most of the red-throated diver records (compare CV map, Figure 5).

**Figure 4:**
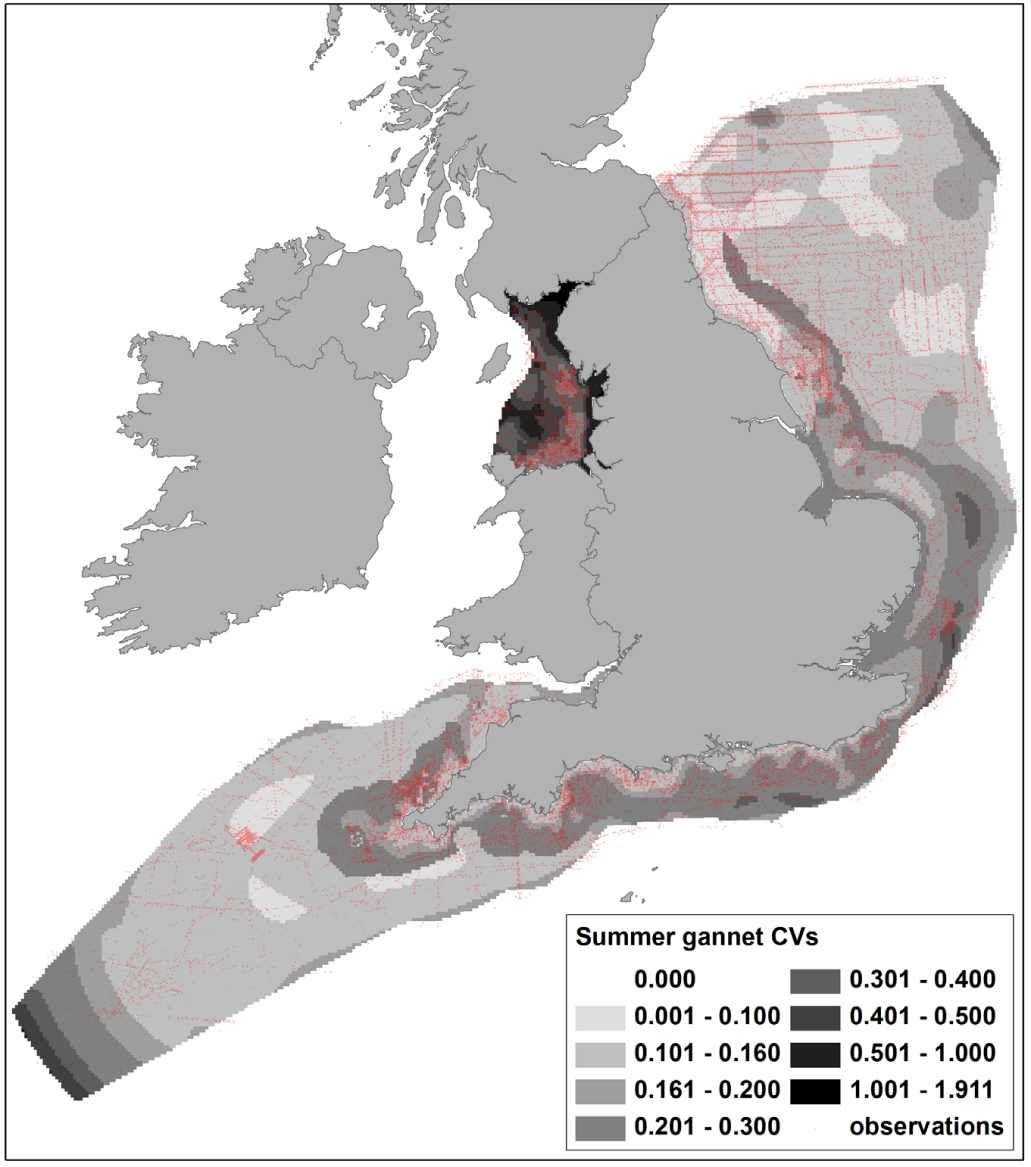
Coefficients of Variation (CVs) of predicted summer densities of gannets from DSM of ESAS boat and WWT Consulting aerial survey data, with survey observations shown. Data with CVs above 0.5 were excluded from sensitivity mapping. Note the generally higher CVs in Liverpool Bay which was modelled separately to the other areas.

**Figure 5:**
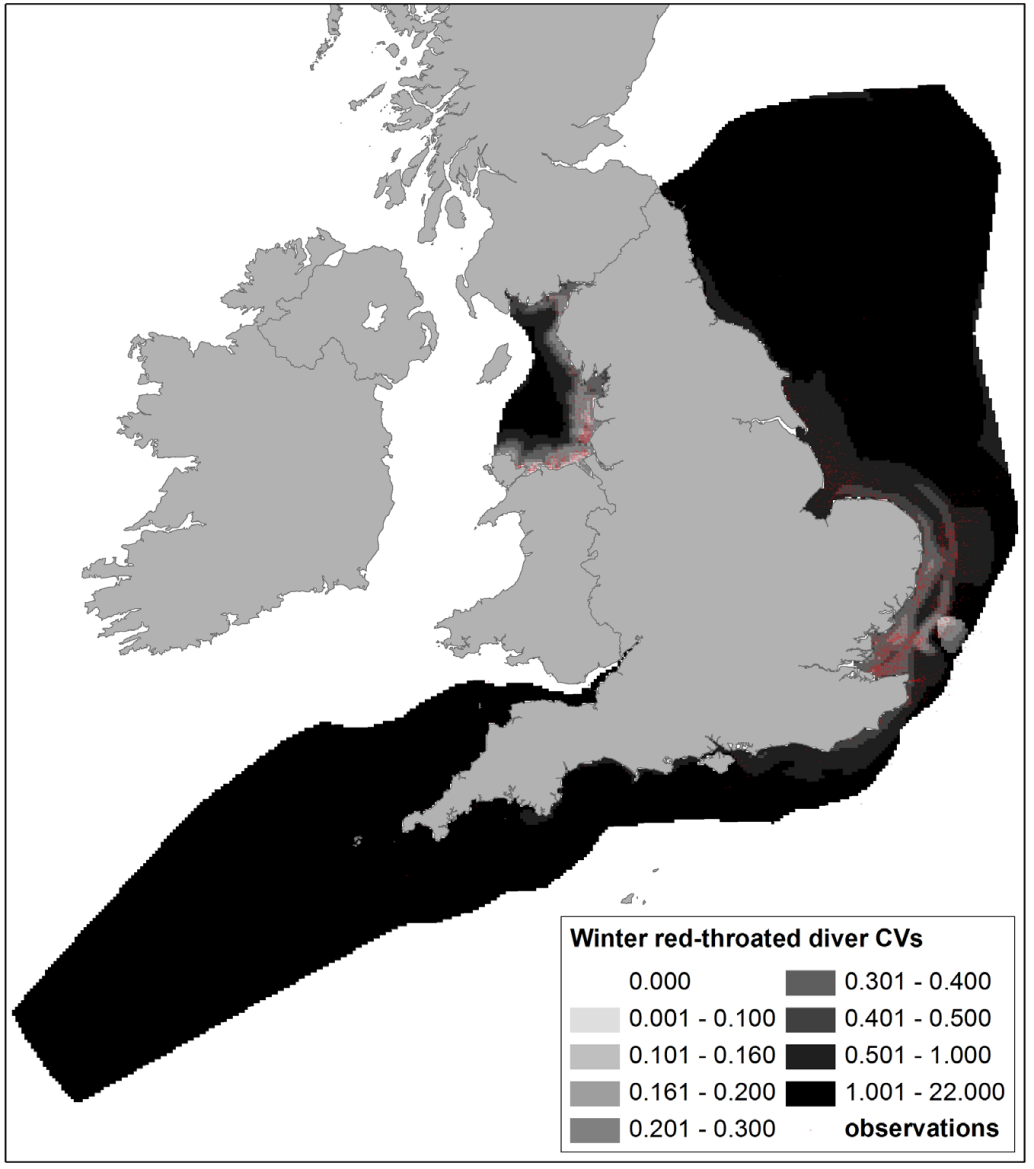
Coefficients of Variation (CVs) of predicted winter densities of red-throated divers from DSM of ESAS boat and WWT Consulting aerial survey data, with survey observations shown. Data with CVs above 0.5 were excluded from sensitivity mapping. Note CVs are considerably higher in the areas not covered by aerial surveys which provided most of the red-throated diver data.

**Figure 6:**
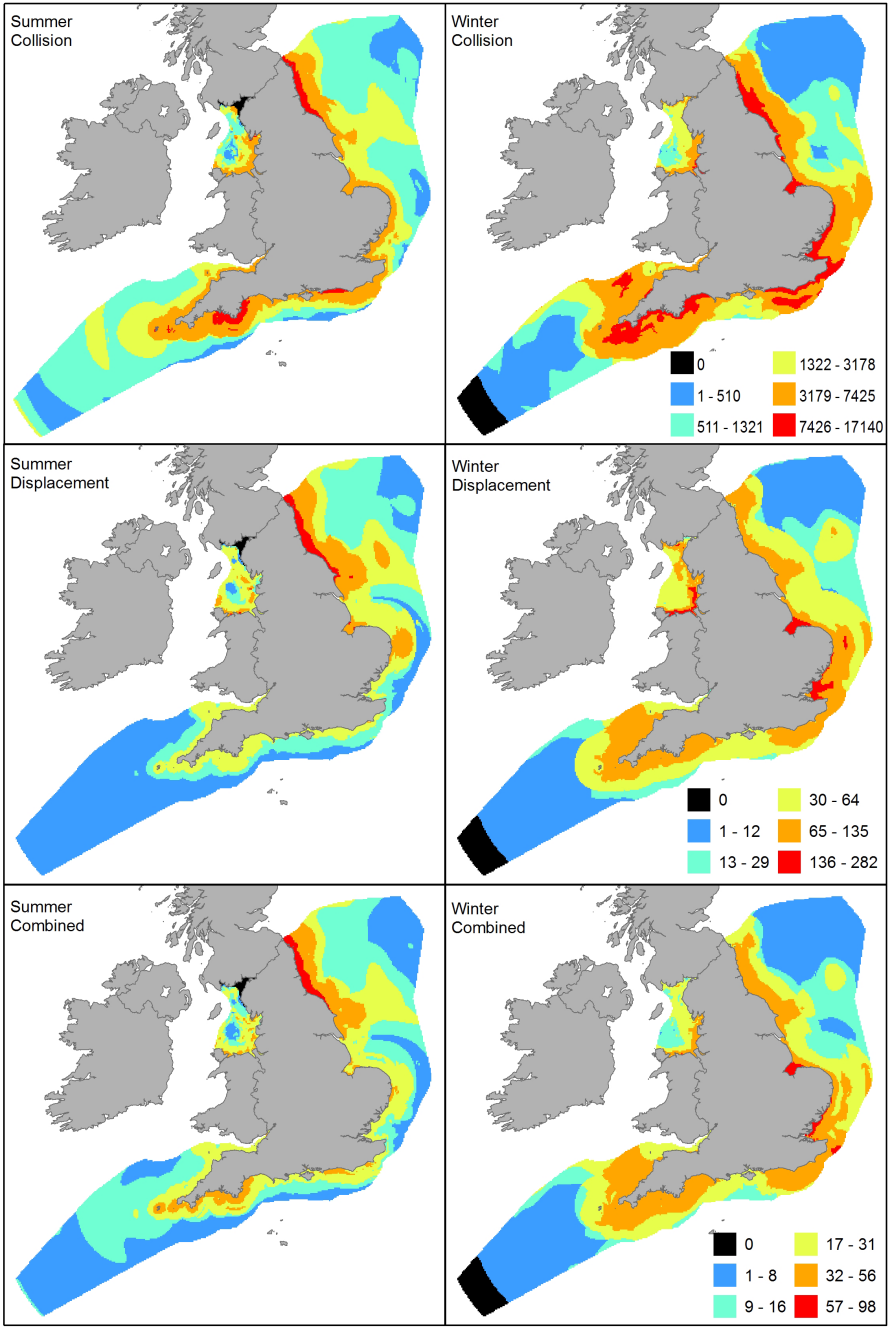
Wind farm sensitivity maps from SeaMaST. The maps were produced by using highest densities from either the boat or aerial density predictions where the CV was less than 0.3 and excluding predictions with CVs higher than 0.5. The natural log of the density (plus one) was then multiplied by each species wind farm collision sensitivity or displacement score and the resulting value summed across species in each 3 km×3 km grid cell. Note where neither dataset had predicted densities with CVs <0.5 the resulting score is exactly zero and highlights areas where across all species coverage and model fits were poor. Summer and winter maps use the same scale.

Table 1 presents the compiled scores for each species sensitivity factor for each species considered. Tables 2 and 3 show the resultant ranked vulnerability scores for collision and displacement impacts that were applied to species to derive sensitivity maps. A sample of the SeaMaST outputs are provided in Figures 1 to 6.

**Table 1:** Scores used in assessing sensitivity of seabird species to collision and displacement/disturbance risks from offshore wind farms in English territorial waters.

Species	Scientific name	a	b	c	d	TotalConservationImportance Score	e	f	g	h	i	j
Greater scaup	*Aythya marila*	1	1	5	3	10	3	4	2	5	4	4
Common eider	*Somateria mollissima*	1	4	4	3	12	2	4	2	3	3	4
Long-tailed duck	*Clangula hyemalis*	1	1	2	3	7	0	3	2	3	3	4
Common scoter	*Melanitta nigra*	2	2	5	3	12	3	3	2	3	5	4
Velvet scoter	*Melanitta fusca*	1	3	4	3	11	3	3	2	3	5	3
Common goldeneye	*Bucephala clangula*	2	2	4	3	11	5	3	2	3	4	4
Red-breastedmerganser	*Mergus serrator*	1	3	1	3	8	5	4	2	2	3	4
Goosander	*Mergus merganser*	2	3	1	3	9	5	4	2	2	4	4
Red-throated diver	*Gavia stellata*	4	3	4	5	16	5	5	2	1	5	4
Black-throated diver	*Gavia arctica*	2	4	4	5	15	5	5	3	1	5	4
Great northern diver	*Gavia immer*	2	4	4	5	15	5	5	2	1	5	3
White-billed diver	*Gavia adamsii*	1	4	1	1	7	5	5	2	1	5	4
Great-crested grebe	*Podiceps cristatus*	2	1	1	3	7	2	4	3	2	3	4
Slavonian grebe	*Podiceps auritus*	3	1	4	5	13	2	4	2	2	3	4
Northern fulmar	*Fulmarus glacialis*	1	5	4	3	13	1	3	2	4	1	1
Cory’s shearwater	*Calonectris diomedea*	1	5	1	5	12	0	3	3	3	1	1
Great shearwater	*Puffinus gravis*	1	5	1	1	8	0	3	3	3	1	1
Sooty shearwater	*Puffinus griseus*	1	5	4	3	13	0	3	3	3	1	1
Manx shearwater	*Puffinus puffinus*	1	5	4	3	13	0	3	3	3	1	1
Balearic shearwater	*Puffinus mauretanicus*	3	4	5	5	17	0	3	3	3	1	1
Wilson’s storm-petrel	*Oceanites oceanicus*	1	4	1	1	7	0	1	3	4	1	1
European storm-petrel	*Hydrobates pelagicus*	1	4	4	5	14	2	1	3	4	1	1
Leach’s storm-petrel	*Oceanodroma leucorhoa*	1	4	4	5	14	2	1	3	4	1	1
Northern gannet	*Morus bassanus*	4	5	4	3	16	12	3	3	2	2	1
Great cormorant	*Phalacrocorax carbo*	2	3	2	3	10	8	4	2	1	4	3
Shag	*Phalacrocorax aristotelis*	3	3	4	3	13	8	3	2	1	3	3
Grey phalarope	*Phalaropus fulicarius*	1	1	1	1	4	10	1	2	2	1	2
Red-necked phalarope	*Phalaropus lobatus*	1	1	5	5	12	10	1	2	2	1	2
Pomarine skua	*Stercorarius pomarinus*	2	4	1	1	8	10	1	5	1	1	2
Arctic skua	*Stercorarius parasiticus*	3	3	5	3	14	10	1	5	1	1	2
Great skua	*Stercorarius skua*	5	4	4	3	16	10	1	4	1	1	2
Long-tailed skua	*Stercorarius longicaudus*	1	4	1	1	7	10	1	5	1	1	2
Sabine’s gull	*Xema sabini*	1	3	1	1	6	20	1	2	2	2	3
Black-headed gull	*Chroicocephalus ridibundus*	5	3	4	3	15	20	1	1	2	2	2
Little gull	*Hydrocoloeus minutus*	2	3	3	5	13	15	1	3	2	1	3
Mediterranean gull	*Larus melanocephalus*	1	3	4	5	13	25	1	2	2	2	2
Common gull	*Larus canus*	5	3	4	3	15	25	1	2	3	2	2
Lesser black-backed gull	*Larus fuscus*	4	5	4	3	16	30	1	2	3	2	1
Herring gull	*Larus argentatus*	5	5	5	3	18	35	2	2	3	2	1
Iceland gull	*Larus glaucoides*	1	5	3	1	10	35	2	2	3	2	1
Glaucous gull	*Larus hyperboreus*	1	5	3	1	10	35	2	2	3	2	1
Great black-backed gull	*Larus marinus*	3	5	3	3	14	35	2	2	3	2	2
Black-legged kittiwake	*Rissa tridactyla*	2	3	4	3	12	15	1	3	3	2	2
Black tern	*Chlidonias niger*	1	4	3	5	13	10	1	4	1	2	3
Little tern	*Sternula albifrons*	3	4	4	5	16	10	1	5	1	2	4
Sandwich tern	*Sterna sandvicensis*	4	4	4	5	17	10	1	5	1	2	3
Common tern	*Sterna hirundo*	2	4	3	5	14	10	1	5	1	2	3
Roseate tern	*Sterna dougallii*	2	4	5	5	16	8	1	5	1	2	3
Arctic tern	*Sterna paradisaea*	1	4	4	5	14	5	1	5	1	2	3
Common guillemot	*Uria aalge*	2	5	4	3	14	1	4	1	2	3	3
Razorbill	*Alca torda*	2	5	4	3	14	0.5	4	1	1	3	3
Black guillemot	*Cepphus grylle*	1	4	4	1	10	0.5	4	1	1	3	4
Little auk	*Alle alle*	1	4	1	3	9	0.5	3	1	1	2	2
Atlantic puffin	*Fratercula arctica*	2	5	4	3	14	0.5	3	1	1	2	3

a = score for highest percent of biogeographic population in England in any season;

b = adult survival score;

c = UK threat status score;

d = Birds Directive score;

e = estimated percentage at blade height;

f = flight manoeuvrability;

g = percentage of time spent flying;

h = nocturnal activity;

i = disturbance susceptibility;

j = habitat specialization.

**Table 2:** Scores for species’ population vulnerability to collision mortality at offshore wind turbines, with species ranked by overall score.

Species	Score for populationvulnerability to collision risk	Classification of risk
Herring gull	1470	Very high
Great black-backed gull	1143	Very high
Lesser black-backed gull	960	Very high
Iceland gull	817	High
Glaucous gull	817	High
Common gull	750	High
Mediterranean gull	542	High
Northern gannet	512	High
Black-legged kittiwake	420	High
Black-headed gull	400	Moderate
Sandwich tern	397	Moderate
Little gull	390	Moderate
Little tern	373	Moderate
Arctic skua	327	Moderate
Common tern	327	Moderate
Great skua	320	Moderate
Roseate tern	299	Moderate
Black tern	260	Moderate
Black-throated diver	225	Moderate
Red-throated diver	213	Moderate
Shag	208	Moderate
Great northern diver	200	Moderate
Red-necked phalarope	200	Moderate
Sabine’s gull	200	Moderate
Great cormorant	187	Low
Pomarine skua	187	Low
Long-tailed skua	163	Low
Arctic tern	163	Low
Common goldeneye	147	Low
Goosander	120	Low
Greater scaup	110	Low
Red-breasted merganser	107	Low
Common scoter	96	Low
White-billed diver	93	Low
Velvet scoter	88	Low
European storm-petrel	75	Low
Leach’s storm-petrel	75	Low
Common eider	72	Low
Slavonian grebe	69	Low
Grey phalarope	67	Low
Great-crested grebe	42	Very low
Northern fulmar	39	Very low
Common guillemot	33	Very low
Razorbill	14	Very low
Atlantic puffin	12	Very low
Black guillemot	10	Very low
Little auk	8	Very low
Long-tailed duck	0	Very low
Cory’s shearwater	0	Very low
Great shearwater	0	Very low
Sooty shearwater	0	Very low
Manx shearwater	0	Very low
Balearic shearwater	0	Very low
Wilson’s storm-petrel	0	Very low

**Table 3:** Scores for English territorial waters marine bird species’ population risk due to displacement by offshore wind farms, ranked by species score.

Species	Overall score forpopulation vulnerabilityto displacement	Classification
Red-throated diver	32	High
Black-throated diver	30	High
Common scoter	24	High
Great northern diver	22	High
Common goldeneye	18	Moderate
Greater scaup	16	Moderate
Velvet scoter	16	Moderate
Slavonian grebe	16	Moderate
Common eider	14	Moderate
Goosander	14	Moderate
White-billed diver	14	Moderate
Little tern	13	Moderate
Common guillemot	13	Moderate
Razorbill	13	Moderate
Great cormorant	12	Moderate
Shag	12	Moderate
Black guillemot	12	Moderate
Red-breasted merganser	10	Moderate
Sandwich tern	10	Moderate
Roseate tern	10	Moderate
Long-tailed duck	8	Low
Great-crested grebe	8	Low
Black tern	8	Low
Common tern	8	Low
Arctic tern	8	Low
Atlantic puffin	8	Low
Black-headed gull	6	Low
Common gull	6	Low
Great black-backed gull	6	Low
Mediterranean gull	5	Very low
Black-legged kittiwake	5	Very low
Sabine’s gull	4	Very low
Little gull	4	Very low
Herring gull	4	Very low
Little auk	4	Very low
Northern gannet	3	Very low
Arctic skua	3	Very low
Great skua	3	Very low
Lesser black-backed gull	3	Very low
Balearic shearwater	2	Very low
Red-necked phalarope	2	Very low
Pomarine skua	2	Very low
Iceland gull	2	Very low
Glaucous gull	2	Very low
Northern fulmar	1	Very low
Cory’s shearwater	1	Very low
Great shearwater	1	Very low
Sooty shearwater	1	Very low
Manx shearwater	1	Very low
Wilson’s storm-petrel	1	Very low
European storm-petrel	1	Very low
Leach’s storm-petrel	1	Very low
Grey phalarope	1	Very low
Long-tailed skua	1	Very low

Summer and winter coverage from boat and aerial surveys shows the largely inshore distribution of aerial surveys compared to the more extensive boat surveys, but also the more patchily distributed boat survey effort (Figure 1). Data allowed density surface maps to be produced for 32 species. Example density surfaces obtained from summing the boat and aerial survey DSM outputs match the pattern of observed data well. Maps for breeding gannets (Figure 2) illustrate a major hotspot south of the Firth of Forth and the large colony at Bass Rock, with other relatively high density areas along the south coast and moderate density areas extending into the North Sea, off Bempton Cliffs. Liverpool Bay supports lower density areas where fewer observations had been recorded. Similarly the density map for wintering red-throated divers (Figure 3) shows highest densities in the sheltered shallow areas of Liverpool Bay, the Outer Thames Estuary and the Wash, matching where the greatest numbers of observations were made.

Closer inspection of Figures 2 and 3 shows apparent anomalies in predicted density. Areas of higher predicted gannet density in the Solway Firth and in the extreme south west do not seem to reflect likely breeding colony distribution [46] [4], whilst higher densities of red-throated divers north of The Wash and along the south coast, with a well-defined hotspot off Cornwall, do not reflect previous knowledge of wintering distribution [6]. However, both areas received little or no survey coverage and so the density predictions may be misleading. Associated maps of confidence are thus crucial in interpretation of the density maps. Figure 4 shows for the gannet density map there is corresponding low confidence (high CVs) in the Solway Firth and extreme south west areas, and reference to survey coverage (Figure 1) shows that this is most likely due to lower effort. Similarly, where large numbers of red-throated divers were recorded and high densities predicted, confidence is relatively high (CVs less than approximately 0.3; Figure 5), but in lower coverage data poor areas, model fits were worse and corresponding CVs were higher. CVs in Liverpool Bay were generally higher than in other areas for most species, due to the comparative lack of data resulting from the geographical separation of models.

By excluding density predictions with CVs of >0.5, data from areas with reasonable coverage and more ‘predictable’ bird distributions were maintained and less reliable predictions excluded from wind farm sensitivity analysis. For example, this approach excluded the predictions of higher gannet densities in the inner Solway Firth, higher red-throated diver densities at locations more than 30 km offshore and unrealistic density estimates for most species from the extreme south west of the study area.

Summer and winter wind farm sensitivity maps for all species combined are shown separately for collision and displacement risks (Figure 6) and also summed by using the highest ranking for each species from either risk. The collision maps indicate areas of higher sensitivity to collision during the summer in the North Sea off north-east England and off the south Devon coast. These result from the presence of higher densities of large gulls which are species which typically exhibit the highest wind farm collision sensitivity scores.

The summer displacement map indicates the coastal waters from Flamborough Head north as the most sensitive area to displacement, a reflection of the higher densities of auks present. More moderate sensitivity areas are found around the coast and extending out to Dogger Bank. In the winter the displacement map is dominated more by the presence of high densities of common scoters and red-throated divers in the Thames Estuary, the Wash and Liverpool Bay.

The overall wind farm summer sensitivity map shows the combined effect of large gulls and auks in the north east (for collisions and displacement respectively) make this the area of highest sensitivity, with more moderate sensitivity around the coast and stretching out to Dogger Bank. In the winter, large gulls, common scoters and red-throated divers in the Wash and inner Thames Estuary and gulls and gannets east of the Kent coast make these the most sensitive areas. Moderately sensitive areas were predicted off the north east and south coasts, and in inner Liverpool Bay. The black areas indicate where no density data could be used for any species as corresponding CVs were greater than 0.5, and thus density and sensitivity predictions are potentially unreliable.

## Discussion

We have successfully produced SeaMaST, a GIS tool that will inform current and future impact assessment and marine spatial planning in England, as well as providing a framework for mapping sensitivity in other geographic areas where there is high demand for wind farms and in other sectors such as wave and tidal energy device deployment, dredging and fishing. As a component of this work, an updated compilation of seabird sensitivity scores in relation to the potential impacts of turbine collision and displacement and disturbance from wind farms has been made to reflect current knowledge.

### Comparison with previous studies

The three main previous seabird density mapping studies covering all or a substantial part of English territorial waters were those by Stone *et al.,* [47] Skov *et al.* [48] and Kober *et al.* [49] [50]. Stone *et al*. used ESAS data from 1980 to 1993, Skov *et al*. used the same data supplemented by data from 1994 and some shore counts and Kober *et al*. used ESAS data from 1980 to 2006. The present study updates these to include ESAS data to 2011 and the WWT Consulting inshore aerial survey dataset. The mapping resolution of this study (3 km×3 km) was substantially finer than that produced by Stone *et al*. (15′ latitude×30′ longitude) due to the use of spatial modelling and four times finer than that used by Kober *et al.* (6 km×6 km), which combined with the associated confidence maps, enables users to assess spatial use over smaller areas. Comparing the predicted density surface maps with the maps produced by Skov *et al.,* Stone *et al.,* and Kober *et al*. showed that for more pelagic species, such as gannets, the distributions and densities produced were similar, though coverage had been increased. The Poisson kriging method used by Kober *et al*. gave more scattered discreet areas of higher density whereas the DSM of this study generally gave wider smooths over areas. However, combining the WWT Consulting aerial surveys with the ESAS data for this study greatly improved coverage for inshore species, such as red-throated divers. The resulting density maps for this species showed generally wider areas of concentrations especially in the Outer Thames Estuary and Liverpool Bay compared with the maps produced by Stone *et al.* [47] and Skov *et al.* [48] with regional abundance estimates comparable to those calculated by O’Brien *et al.* [6].

The study updated previous work on seabird sensitivity to wind farms in Scottish waters [14] [37] to include more recent demographic data and defining populations relevant to English territorial waters. This enables comparison to be made of relative species sensitivity in English and Scottish waters.

### Methodological considerations

The method of wind farm sensitivity scoring used has built on previous peer-reviewed approaches, most notably those of Garthe and Hüppop and Furness *et al*. [13] [14], to produce species rankings and relative scores separately for collision and displacement impacts. Methodological considerations have been discussed previously in those publications, but include the accuracy of flight height data, which is weighted as the most important factor in collision risk sensitivity scoring, and the recorded displacement of species from wind farms. Data informing both of these factors are sparse and inconsistent, relating to the difficulty of recording these accurately at sites [37]. Similarly, though there is a relatively well-used framework for relating collision mortality to impacts on populations through reference to Population Viability Analysis (PVA) [51] and Potential Biological Removal (PBR) studies [52], the impacts on populations through displacement/disturbance are poorly understood. The complicating issue of macro-avoidance was partially examined here, concluding that insufficient data were presently available for most species to reliably assess the degree of attraction (and thus potentially increased collision risk) or avoidance from some distance away from the wind farm (and thus suppressed collision risk). Not including a measure of macro-avoidance had the effect of assuming all species exhibited this behaviour to the same degree. The issue of macro (and micro) avoidance has deservedly attracted a lot of attention and funding (e.g. through the Offshore Renewables Joint Industry Programme [53]) as it is integral to Collision Risk Modelling carried out as part of wind farm assessments [54]. As data from these projects become available it may be possible to include relative macro-avoidance rates in future species sensitivity scoring.

In undertaking an analysis of such large marine bird datasets, a number of challenges were encountered. Extracting the data from different sources identified the diversity in which project specific data were reported. A recommendation is to use a common standard database template and adoption of agreed species groups categories for species that could not be identified in the field. The ESAS database standard [55] is obviously well reasoned however the adoption of new protocols, such as continuous boat-based recording and the collection of other data such as flight height, seconds of time of observations and differences in recording sighting conditions cannot be accommodated in the current standard format. Agreement should be sought on an updated format where any potentially valuable data can be preserved. Increasingly, digital imaging methods are being used for marine surveys [56] and if they are to be incorporated in a common dataset format in the future there will need to be provision for their particular metadata requirements also, such as altitude, ground sample distance, camera configuration and sampling protocol.

In modelling the data the relative importance of the different spatial scales of survey coverage were made obvious. The high intensity aerial surveys provided robust estimates of inshore species in relatively small coastal features, but estimates quickly became more unreliable moving further offshore away from the covered areas. By contrast the generally more extensive boat surveys enabled more confident predictions to be made across larger areas as they encompassed a wide spread of samples and covariates to model, though any finer scale patterns may have remained undetected. This will be relevant in planning further survey or monitoring work so that data best inform the objectives. Reference to the confidence maps in SeaMaST may also help inform where survey effort may best be applied to improve modelling.

Overall where there was good spatial coverage of surveys the DSM predictions produced had high confidence and matched the patterns of survey observations. Further from these areas of good coverage the predictions were more dependent on the modelling of covariates and confidence in the predictions tended to diminish. For this reason, in compiling the sensitivity maps, data were excluded if CVs exceeded a threshold value, so only the more reliable data were used as evidence. In interpreting results however it should be noted that data exclusion does not mean birds were not recorded there, or that they would not be recorded if they were surveyed.

It is hoped that, where they become available, other datasets, such as from Round 3 wind farm sites, can be appended in subsequent updates to the tool. Work is currently underway using spatial modelling to produce density surfaces from the ESAS and WWT Consulting aerial databases for seabird species in Welsh, Scottish and Northern Irish territorial waters.

### Use of SeaMaST

SeaMaST has been made available to UK marine planning practitioners and hopefully by publication of this paper will be made more widely available through the Natural England website (www.naturalengland.org.uk). It is intended that SeaMaST will be used in a variety of ways by numerous interested parties. As well as informing Marine Spatial Plans being drawn up for England [57], SeaMaST should provide a template for other countries needing to understand seabird sensitivity in a marine planning context. It may also be useful in contributing to evidence informing new Marine Protection Areas for seabirds, as well as identifying those areas requiring future monitoring effort.

Additionally, SeaMaST can be used to inform various stages of Environmental Impact Assessment in the marine environment. For instance, it can provide information on existing knowledge of seabird distribution, and the confidence associated with that knowledge, to tailor baseline monitoring plans to answer specific questions; it can provide a ‘sense check’ to predictions made in assessments; and it can be used by decision makers to contextualise the significance of potential impacts. Furthermore, we anticipate SeaMaST will be used for individual or institutional research requiring seabird distribution maps or ecological sensitivity to renewables development.

Finally, we intend to develop SeaMaST to reflect new data emerging over time. This will allow updating of base maps of seabird distribution, as well as permitting additional layers of sensitivity to be created. This process involves the identification of potential sensitivities (e.g. to bycatch from fisheries [58]) and then the creation of SSIs. These can then be applied to the seabird distribution maps to produce sensitivity maps for a range of pressures and threats, which may be useful for reporting into schemes such as the EU Marine Strategy Framework Directive.
