# Correction: Mapping Seabird Sensitivity to Offshore Wind Farms

**DOI:** 10.1371/journal.pone.0170863

**Published:** 2017-01-23

**Authors:** Gareth Bradbury, Mark Trinder, Bob Furness, Alex N. Banks, Richard W. G. Caldow, Duncan Hume

The Data Availability statement for this paper is incorrect. The correct statement is: The authors confirm that all data underlying the findings are fully available without restriction. For European Seabirds At Sea data, contact JNCC (http://jncc.defra.gov.uk/page-4469); for aerial survey data, contact WWT (http://www.wwtconsulting.co.uk/our-services/ecological-surveys-and-assessment/aerial-surveys-for-seabirds-and-cetaceans/). SeaMaST is available from Natural England (http://www.gis.naturalengland.org.uk/pubs/gis/GIS_register.asp) or from the Natural England GI Data Manager: NaturalEnglandGIDataManagers@naturalengland.org.uk.
